# The Impact of Perceptual/Concurrent and Mnemonic Digits on Temporal Processing: A Congruency Effect of Numerical Magnitudes

**DOI:** 10.3389/fpsyg.2016.02014

**Published:** 2017-01-09

**Authors:** Zhao Fan, Guomin Jing, Xianfeng Ding, Xiaorong Cheng

**Affiliations:** ^1^Key Laboratory of Adolescent Cyberpsychology and Behavior, Central China Normal University (CCNU), Ministry of EducationWuhan, China; ^2^School of Psychology, Central China Normal University (CCNU)Wuhan, China; ^3^Department of Education, Taiyuan Normal UniversityTaiyuan, China

**Keywords:** perception, memory, digits, number-time association (NTA), congruency effect

## Abstract

Task-irrelevant stimulus numbers can automatically modulate concurrent temporal tasks——leading to the phenomenon of number-time association (NTA). Recent research provides converging evidence that the NTA occurs at the stage of temporal memory. Specifically, a reference memory containing encoded duration information can be modified by perceptual/concurrent digits, i.e., a perceptual/concurrent digit-induced NTA. Here, with five experiments, we investigated whether another working memory (WM)-related mechanism was involved in the generation of NTAs and how this memory-induced NTA was related with the perception-induced NTA. We first explored whether similar NTA effects existed for mnemonic digits which disappeared before time encoding but were actively maintained in WM, i.e., a mnemonic digit-induced NTA. Experiments 1–3 demonstrated both types of NTAs. Further, we revealed a close relationship between the two types of NTAs in two contexts. First, the mnemonic digit-induced NTA also relied on a perceptual number-time co-occurrence at time encoding. We found that the mnemonic digits influenced subsequent temporal processing when a task-irrelevant constant number ‘5’ was presented during target encoding, but not when a non-numerical symbol was presented, suggesting that temporal representations in the reference memory could be accessed and modified by both sensory and postsensory numerical magnitudes through this number-time co-occurrence. Second, the effects of perceptual and mnemonic digits on temporal reproduction could cancel each other out. A congruency effect for perceptual and mnemonic digits (relying on memorization requirement) was demonstrated in Experiments 4 and 5. Specifically, a typical NTA was observed when the magnitudes of memorized and the perceptual/concurrent digits were congruent (both were large or small numbers), but not when they were incongruent (one small and one large numbers). Taken together, our study sheds new light on the mechanism of NTA.

## Introduction

Time plays a critical role in our daily cognition. It has been well-established that temporal processing^1^ is influenced by temporal as well as non-temporal information (e.g., Fortin et al., 1993; Brown, 1997; Mattes and Ulrich, 1998; Oliveri et al., 2008; Eagleman and Pariyadath, 2009). Specifically, various non-temporal, magnitude dimensions, such as stimulus size, number of contained items, intensity and speed, exert critical impact on perceived durations (Berglund et al., 1969; Mo and Michalski, 1972; Dormal et al., 2006; Kanai and Watanabe, 2006; Xuan et al., 2007; Alards-Tomalin et al., 2014; Rammsayer and Verner, 2014).

As an important form of magnitude, numbers in the notation of Arabic digits have also been found to have an effect on temporal processing. For example, in a temporal discrimination task, numerical magnitudes can be activated automatically—leading to a subjective time expansion for stimuli with a large number (e.g., 9) and a time compression for stimuli with a small number (e.g., 1), even though the magnitude information was task-irrelevant (Xuan et al., 2007; Oliveri et al., 2008; Vicario et al., 2008). This phenomenon, known as number-time association (NTA) (Bi et al., 2014), has also been found in time reproduction tasks (Chang et al., 2011; Cai and Wang, 2014).

Traditionally, temporal processing is regarded as a multiple stage processing, including time encoding- extracting temporal information, time memory- holding the perceived time into memory, and time decision- making a temporal judgment for a response (Gibbon et al., 1984; Wearden, 1999). In a typical temporal reproduction task, a pacemaker-counter component exists to produce and store the internal representation of a target interval. A memory component, i.e., a reference memory (Zakay and Block, 1997), is necessary to hold the number of pulses from the counter after the time encoding stage in order to provide a durable internal representation of the target duration. Further, a decision-making component (Yates et al., 2012) is critical to compare the retrieved duration representation of the target from the reference memory with the accumulated pulses during the reproduction. A stop signal is triggered by the decision-making component to stop the reproduction process.

An earlier study (Chang et al., 2011) suggested that task-irrelevant magnitude affected temporal processing at the encoding stage. By using a temporal reproduction task with a key-press response, Chang et al. (2011) found a typical NTA pattern when the perceptual/concurrent digits appeared during the standard intervals, but an opposite NTA pattern when the digits appeared during the reproductions, implying a time encoding account for the NTA. Recently, the time encoding account for the NTA has been challenged by an alternative explanation, i.e., a time memory-based account for the NTA. Particularly, the inversed NTA pattern found by Chang et al. (2011) was not observed in follow-up studies with similar reproduction paradigms, though the typical NTA pattern was very stable when the perceptual/concurrent digits appeared during the standard intervals. For example, Cai and Wang (2014) found a typical NTA when the numbers were presented for a standard duration in a temporal reproduction paradigm. The reproduced duration was longer when the standard stimulus was a large number (e.g., 8 or 9) than when it was a small number (e.g., 1 or 2). However, in contrast to Chang et al. (2011), they did not find an inversed NTA pattern when the standard durations were occupied by a green dot and the numbers were presented during time reproductions. Instead, the numerical magnitudes did not influence temporal processing in this condition. Given that the encoded duration generated by online key-pressing was not yet been held into memory during time reproductions, Cai and Wang (2014) proposed that numerical magnitudes affected temporal processing at the stage of time memory, rather than time encoding. In another example, Rammsayer and Verner (2015) drew the same conclusion using a similar paradigm with different stimuli, i.e., black circles in different sizes. They found that reproduced durations were modulated by sizes of stimuli only when stimulus sizes varied during time perception but not during time reproduction. Taken together, these results provide converging evidence to support the view that the NTA occurs at the stage of temporal memory. In other words, the reference memory containing the representation of the already-encoded target duration can be modified by perceptual/concurrent digits.

The above studies demonstrated that numerical magnitudes in the form of Arabic digits appearing during time encoding can influence temporal processing and produce a typical NTA, i.e., a perceptual/concurrent digit-induced NTA. A recent study (Vicario, 2011), using a temporal bisection task, highlighted the critical role of magnitude contextual factors, such as relative size differences of the displayed numbers within the same block, in the generation of a NTA. In this study, Vicario (2011) asked participants to time the duration of each test cue, consisting of a numeral out of 1, 2, 8, and 9, and perform a temporal midpoint judgment task by pressing a response key. A typical NTA was found when numbers were all randomly arranged in the same block (intermingled design) only, but not when numbers were blocked according to their sizes (blocked design). Vicario (2011) proposed that participants performed implicit comparisons of size differences across the displayed numbers in the intermingled condition. This proposal implied a potential role of a mnemonic system in the generation of NTA since the magnitude representations of the previous trials within the same block were likely to be kept temporarily in a mnemonic system of the brain, such as working memory (WM), before being compared with the magnitude representation of the current trial. Since the role of WM in NTAs was hypothetical and there was no memory requirement for the paradigm used in Vicario’s (2011) study, it would be interesting to carry out a study with explicit WM manipulation to explore the potential role of WM in NTAs directly. Thus, in the present study we explored whether a similar NTA effect existed when digits disappeared before time encoding but were maintained in the WM in an active and explicit way, i.e., a mnemonic digit-induced NTA. In other words, we investigated whether the number-time co-occurrence at the stage of time encoding was necessary for an NTA. Will a digit-induced numerical magnitude which is out of perception and kept in WM explicitly distort subsequent temporal reproduction even when a mnemonic digit and a standard duration are presented asynchronously?

This question has significance for at least three reasons. First, answers to this question can extend our understanding of the mechanism of the perceptual/concurrent digit-induced NTA. For this type of NTA, it is not yet clear whether the accessibility of the task-irrelevant numerical information to the reference memory is triggered directly by a perceptual presentation of the digit itself or alternatively by a postsensory processing after time encoding, such as an automatically activated mnemonic reminiscence of the digit. If the latter case is true, we would expect that a new paradigm where digits are actively maintained in the WM before time encoding will show a similar phenomenon of NTA, i.e., a mnemonic digit-induced NTA. On the other hand, if the former case is true, mnemonic digits in the new paradigm will not automatically induce a typical NTA.

Second, recent studies (Pan and Luo, 2011; Bi et al., 2014) revealed that contents of WM can modulate concurrent time processing. For example, Pan and Luo (2011) asked participants to memorize the color of a stimulus before performing a temporal discrimination task and found that a task-irrelevant color influenced time perception. More specifically, they showed that standard durations were overestimated when colors in WM and those in temporal discrimination were matched. This study suggested that WM plays an important role in time estimation. Thus, it would be interesting to ask further whether magnitude information in the notation of Arabic numbers exerts influence on temporal processing. Indeed, a recent study (Bi et al., 2014) investigated this issue. In their design, a dual task paradigm combining a comparative duration judgment and a number memorization was used. Participants were required to memorize a digit and then make a decision on which one of the two successive digits was presented longer or shorter. Using accuracy as the dependent variable, they found longer perceived durations were related to larger perceptual/concurrent digits and shorter perceived durations related to smaller perceptual/concurrent digits, i.e., a typical perceptual/concurrent digit-induced NTA in a temporal comparison task. Interestingly, this NTA was observed only when the contents in WM mismatched the stimuli in the temporal comparison judgment, i.e., both digits for the temporal comparison were different from the memory digit. In contrast, this NTA was abolished when one of the digits for the temporal comparison was the same as the memory digit. This result supported a view that perceptual/concurrent digit-induced NTAs in a temporal comparison task can be disrupted by the contents of WM (Bi et al., 2014).

However, some technical aspects of Bi et al. (2014) study motivated us to set up a new paradigm in the present study. Bi et al. (2014) only explored the modulation of WM on NTAs when a mnemonic digit and one of the two perceptual/concurrent digits were the same (matched) or not (unmatched). Their manipulation could not distinguish whether the matches were at a numerical magnitude level or at a graphemic level. Thus, it is still not clear whether Arabic digits maintained in WM can modify the NTA at a level of magnitude when the two digits were different (graphemically unmatched) but magnitudely congruent (both were large numbers or small numbers) or incongruent (one small and one large number). This question has specific implications and significance in the light of earlier research indicating that digits of similar magnitude, such as 1 and 2 or 8 and 9, produce qualitatively similar effects during the activation of the NTA (Cai and Wang, 2014) or the space-number association (Dehaene et al., 1993). Moreover, Bi et al. (2014)’s study used a comparative duration judgment which debatably suffered from a decision bias according to a previous study (Yates et al., 2012). With two experiments, Yates et al. (2012) demonstrated that the effect of stimulus size on time estimation actually depended on which type of psychophysical measurement was used. In their first experiment, a NTA-like interference of size on time, i.e., larger stimuli were judged to last longer, was observed when comparative judgments were used (i.e., to judge which one of two stimuli was presented longer). However, when equality judgments were used in a second experiment (i.e., to judge whether two stimuli were presented for the same duration or not), an unexpected inversed NTA-like interference of size on time, i.e., larger stimuli were judged to last shorter, were observed instead. Yates et al. (2012) proposed that the NTA-like interference of size on time in their study was not necessarily due to that the perceived durations were effectively changed by stimulus sizes. Instead, it was very likely that the stimulus size simply biased decisions about duration, especially when a task relying on a comparative judgment was used. Thus, in the present study it might be necessary to setup a new paradigm with a temporal reproduction task which is free of the potential decision-making bias compared with a temporal comparative task (Rammsayer and Verner, 2014). Another rationale of the new paradigm is to see whether NTAs can be held by replacing the time comparison task with a time reproduction task.

The third reason for us to explore the existence of a mnemonic digit-induced NTA is based on many previous studies (Chacones and Nashman, 1989; Owen and Evans, 1996; Herwig et al., 2007) indicating qualitatively different mechanisms between the perceputal/concurrent, sensory-based processing and the mnemonic, postsensory-based processing. For example, Sheth and Shimojo (2003) demonstrated that visual context, such as surround motion, may have similar or opposite effects on spatial perception and spatial WM, depending on the strength of the stimulus. Other studies also found strong interactions beween perception and WM (Gao et al., 2011; Olkkonen and Allred, 2014). Thus, by comparing the potential effect of a mnemonic digit-induced NTA with that of a perceptual/concurrent digit-induced NTA, the present study may shed some light on the dynamics between perceptual and mnemonic processing. Here, it is noteworthy that we adopted operational definitions of ‘perception’ and ‘memory’ from Sheth and Shimojo (2003, p. 173)’s study, i.e., “Operationally, perception can be regarded as a representation in the brain of something currently or very recently present on the retina, and memory (disregarding otherwise important distinctions between different kinds of memories) as an internal representation of something that was shown in the past.”

The present study tried to explore the impact of perceptual/concurrent and mnemonic digits on temporal processing in a series of experiments. Particularly, we aimed to explore the existence of a mnemonic digit-induced NTA and how it might be related with a perceptual/concurrent digit-induced NTA. According to the above-mentioned operational definitions, the ‘perceptual/concurrent’ refers to the online sensory process during time encoding of a target interval in a temporal reproduction task and the ‘mnemonic’ refers to memorization process well before the time encoding.

## General Methods

**FIGURE 1:**
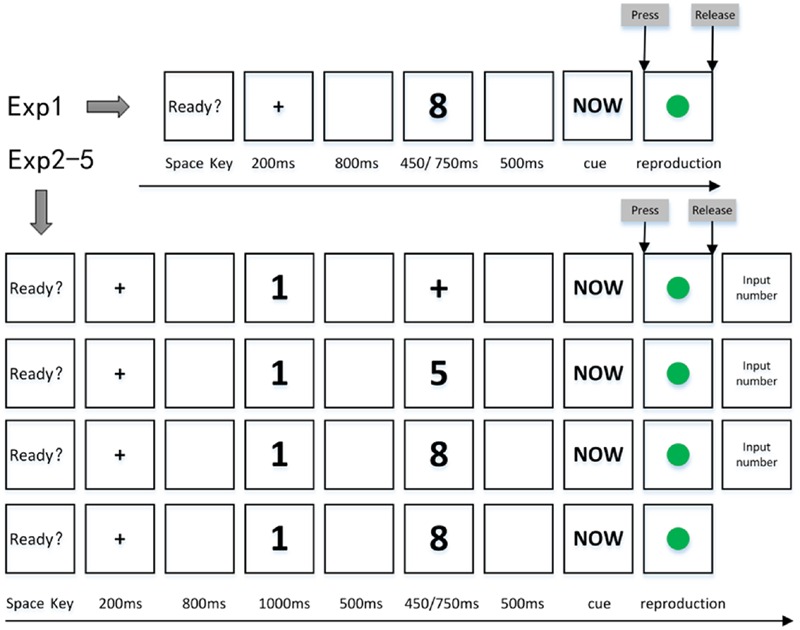
**Exemplary trial sequences for Experiments 1–5.** The dot was green in these experiments. **Upper panel** is for Experiment 1 (top row in the figure): following a fixation cross, a perceptual/concurrent digit (1, 2, 8, or 9) was presented for a standard duration of either 450 ms (short) or 750 ms (long). Participants were instructed to reproduce the standard duration containing the perceptual/concurrent digit by keeping the ‘0’ key pressed following they saw the cue word “NOW.” **Lower panel** is for Experiments 2–5 (from the second row to the fifth row in the figure): the procedure was almost the same as Experiment 1, except that after the fixation cross, a mnemonic digit (1, 2, 8, or 9) was present for 1000 ms. Following the mnemonic digit, a standard stimulus (a black cross in Experiment 2, a constant number 5 in Experiment 3 and one of the number in 1, 2, 8, and 9 in Experiments 4–5, see **Table 1**) was presented for 450 ms (short) or 750 ms (long). At the end of the trial, participants were instructed to reproduce the standard duration and to input the mnemonic digit (except in Experiment 5) after time reproduction.

Five experiments were conducted in the present study. The paradigm used in each experiment is summarized here. Experiment 1 used a time reproduction task (see the first row of **Figure 1** and **Table 1**) aiming to replicate the perceptual/concurrent digit-induced NTA (Chang et al., 2011; Cai and Wang, 2014). Two levels of target duration (short vs. long) were provided to check whether the NTA relied on the length of the target presentation because there were mixed results in previous studies upon this point (Bi et al., 2014; Cai and Wang, 2014, see General Discussion for more on this issue). Experiments 2 and 3 were carried out to explore the existence of a mnemonic digit-induced NTA with a dual task paradigm. Experiment 2 required participants to memorize a briefly presented Arabic digit (mnemonic digit) and keep it in WM before encoding a target duration containing a non-numerical symbol, e.g., a black cross, on the display (see the second row of **Figure 1** and **Table 1**). A slightly different paradigm was used in Experiment 3 to explore this issue when the black cross was replaced by a task-irrelevant constant number ‘5’ (see the third row of **Figure 1** and **Table 1**). Taken together, Experiments 2 and 3 can provide information on whether a mnemonic digit can exert a direct influence on the subsequent time reproduction (in Experiment 2) or a mnemonic digit-induced NTA might rely on a perceptual number-time co-occurrence at time encoding (in Experiment 3). Experiment 4 aimed to explore the dynamic interactions between the two types of NTAs. In order to accomplish this goal, two types of digits, a mnemonic digit and a perceptual/concurrent digit, were used. Participants were required to memorize a number (mnemonic digit) first and then reproduce a target duration containing a perceptual/concurrent digit by key-pressing responses (see the fourth row of **Figure 1** and **Table 1**). We were interested in the fate of the NTA effect when the perceptual and mnemonic digits were in different magnitudes (incongruent condition: one number was small and the other was large), and when both digits were of similar magnitude (congruent condition: both were large or small numbers). This is a novel paradigm compared with the ones used in previous literature (Chang et al., 2011; Bi et al., 2014; Cai and Wang, 2014) by combining the procedure of Experiment 1 aiming to explore the perceptual/concurrent digit-induced NTA and that of Experiment 3 aiming to explore the mnemonic digit-induced NTA. Experiment 5 was a control experiment, aiming to explore whether the memory maintenance was a prerequisite for the effect found in Experiment 4 (see below for more details) when the mnemonic requirement was removed (see the fifth row of **Figure 1** and **Table 1**). The main differences of stimulus presentations and task requirements among the paradigms in Experiments 1–5 of the present study and the three paradigms in previous studies (Chang et al., 2011; Bi et al., 2014; Cai and Wang, 2014) are summarized in **Table 1**.

**Table 1:** Stimulus and task differences among the paradigms in Experiments 1–5 of the present study and the three paradigms in previous studies.

Experiment/paradigm	Mnemonic digits	Stimulus 1	Stimulus 2	Temporal response	Memory test
Experiment 1	No	1, 2, 8, or 9	No	Reproduction	No
Experiment 2	Yes	“+”	No	Reproduction	Input number
Experiment 3	Yes	5	No	Reproduction	Input number
Experiment 4	Yes	1, 2, 8, or 9	No	Reproduction	Input number
Experiment 5	Yes	1, 2, 8, or 9	No	Reproduction	No
Cai and Wang, 2014	No	1, 2, 8, or 9	No	Reproduction	No
Chang et al., 2011	No	1, 2, 8, or 9	No	Reproduction	No
Bi et al., 2014	Yes	1, 2, 8, or 9	1, 2, 8, or 9	Comparison	Number recognition

The 5 experiments in the present study differed in both stimulus presentation and task requirements. A mnemonic digit was presented in each trial of Experiments 2–5 but not in Experiment 1. Participants were required to remember and input the mnemonic digit in Experiments 2–4 but not in Experiment 5. The contents of the standard stimulus were also different across the five experiments, i.e., one of the numbers from “1, 2, 8, and 9” for Experiments 1, 4, and 5, “+” for Experiment 2, and “5” for Experiment 3. The tasks in Chang et al. (2011) and Cai and Wang (2014) involved no mnemonic digits and memory requirements, comparing the tasks in Experiments 2–4 of the present study. Bi et al. (2014) used a time comparison task, whereas time reproduction tasks were used in Experiments 2–4 of the present study.

Participants from the Central China Normal University (CCNU) were recruited and paid for participating the five experiments in this study. All participants in all experiments of this study were right-handed, with normal or corrected-to-normal vision, and were naive to the purpose of the study. The present study was approved by the Ethics Committee of the CCNU. All participants in the present study signed a consent form before participation. Each participant was asked to complete fewer than 16 practice trials before the formal test. Participants were tested individually in a quiet testing room and seated in front of a monitor with their left index finger on the ‘space’ key and right index finger on the ‘0’ key. The viewing distance was about 57 cm. Stimuli were presented on a 17″ CRT monitor with a 1024 × 768 resolution at a refresh rate of 100 Hz. The stimuli were black numbers (1.5° × 1.2°) or a black cross (1.5° × 1.5°) on a white background and were presented in the center of the monitor. Psychophysics Toolbox (Brainard, 1997; Pelli, 1997) implemented on MATLAB (MathWorks Inc.) was used to generate stimuli and run all the experiments.

## Experiment 1: The Effect of Perceptual/Concurrent Digits – A Typical NTA

### Methods

#### Participants

Twenty (18 females and 2 males; mean age 20.1) were recruited and paid for Experiment 1.

#### Stimuli and Design

In this experiment, stimuli could be one of four numbers (1, 2, 8, or 9), which were separated into two categories based on their magnitudes: small numbers (1 and 2) and large numbers (8 and 9). There were two standard durations for the perceptual/concurrent digits which were also separated into two categories: short (450 ms) or long (750 ms). Each block of the experiment contained 2 repetitions of eight combinations of the perceptual/concurrent digits (1, 2, 8, or 9) and standard durations (450 ms or 750 ms). The order of trials was randomized within a block. There were 10 blocks in this experiment, resulting in 160 trials in total. Participants took a rest after every two blocks.

#### Procedure

Each trial began with the presentation of a prompting sentence (“If you are ready, press the ‘space’ key to continue!”). A fixation cross (0.7° × 0.7°), lasting for 200 ms after participants’ key pressing, and was followed by a blank screen for 800 ms. Then a digit was presented for one of the two standard durations. After the offset of the standard duration, the word “NOW” was shown on the screen as a prompt for participants to start reproducing the standard duration. In order to make sure that participants clearly knew when the standard duration ended, a 500 ms blank screen was placed between the standard duration and the cue word. Participants reproduced the standard duration by holding down the “0” key on the keyboard. The “0” key pressing terminated the cue word immediately and then a green dot (1.5° × 1.5°) was displayed on the screen center. The presentation of the green dot was contingent on the continuous “0” key pressing and stopped when the “0” key was released. A prompting sentence for the next trial was shown on the screen immediately after participants released the “0” key (see the first row of **Figure 1**).

### Results and Discussion

The same trimming method as that used in Chang et al. (2011), was adopted to exclude outliers in duration reproductions. Reproductions of the standard durations that were shorter than 1/3 of the standard durations or longer than three times of the standard durations were excluded. These criteria led to exclusion of 0.531% total trials from the collected data. A similar approach was also used in a recent study, i.e., Cai and Wang (2014). The justification for using this approach rather than a standard deviation (SD)-based one was indicated in Cai and Wang (2014, p. 4), i.e., “We adopted such a trimming method rather than one based on SD because the latter method, due to the right-tailed distribution of time reproduction data, tends to include very brief reproductions caused by accidental key presses/releases (e.g., 2 or 3 SDs away from the mean would often result in a negative score)”.

A 2 × 2 repeated-measures analysis of variance (ANOVA^2^) was conducted with magnitude of the perceptual/concurrent digits (small vs. large) and duration (short vs. long) as independent variables and reproductions of the standard durations as the dependent variable. The results revealed a significant main effect of standard durations [*F*(1,19) = 68.735, *p* < 0.001, η^2^ = 0.783], indicating that shorter standard durations were reproduced significantly shorter than longer standard durations (493 ± 107 ms vs. 636 ± 135 ms in a format of mean value of the reproduced duration ± SDs; the same format is used throughout the present study when mean reproduced durations are referred to). Critically, the main effect of numerical magnitude was also significant [*F*(1,19) = 7.222, *p* = 0.015, η^2^ = 0.275]. The standard durations with large numbers were reproduced longer than those with small numbers (571 ± 119 ms vs. 559 ± 113 ms). The interaction between numerical magnitude and standard duration was also significant [*F*(1,19) = 7.325, *p* = 0.014, η^2^ = 0.278]. That is, standard durations with larger numbers were reproduced longer than those with smaller numbers when the standard durations were long (625 ± 143 ms, difference = -23 ms, *p* = 0.002), but not when the standard durations were short (*p* = 0.667). The main effect of numerical magnitude, and more importantly the simple effect of numerical magnitude at the level of long standard duration, demonstrated that numerical magnitude affected temporal processing, i.e., a typical perceptual/concurrent digit-induced NTA pattern.

**FIGURE 2:**
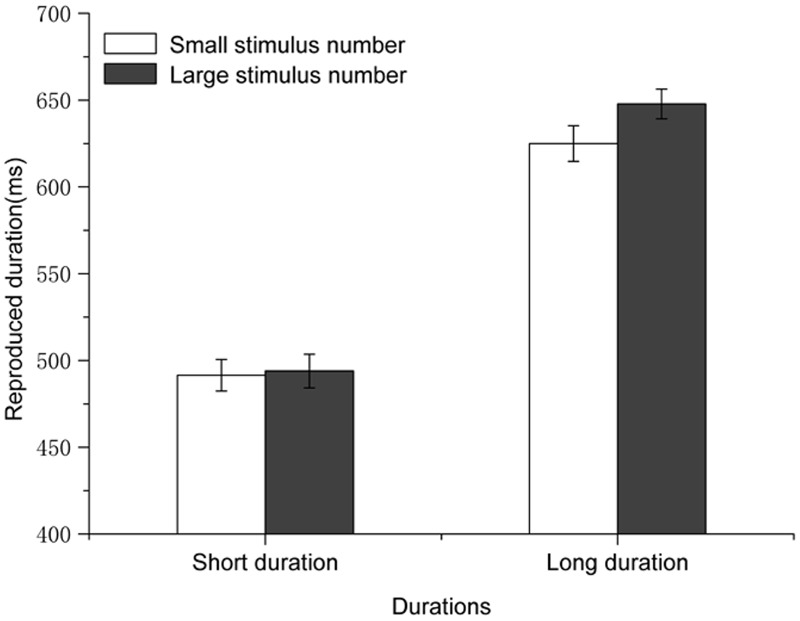
**Results for Experiment 1: the standard durations with large perceptual/concurrent numbers were reproduced longer than those with small perceptual/concurrent numbers, and this effect was only observed when the standard durations were long.** Error bars are within-subjects SEs (Cousineau, 2005).

We successfully replicated the classic perceptual digit-induced NTA with a temporal reproduction task in Experiment 1. Standard durations with large numbers (8 or 9) were reproduced longer than those with small numbers (1 or 2). This result is consistent with previous studies (Chang et al., 2011; Cai and Wang, 2014). Particularly, the magnitude of the perceptual/concurrent digit-induced NTA in the present study (Mean difference of time reproductions between large and small digits = 12 ms) was in the same range as that of a previous study (Cai and Wang, 2014, Experiment 1, Mean difference of time reproductions between large and small digits = 13 ms) with a similar paradigm. Our data in Experiment 1 suggest that numerical magnitudes could modulate perceived durations, and this effect was subject to the interaction between numerical magnitude and standard duration (see **Figure 2**). Such an interaction was not observed in the previous related studies using the same standard durations (Chang et al., 2011; Cai and Wang, 2014). However, the fact that standard durations modulated a NTA was also observed in a recent study (Rammsayer and Verner, 2015), which is consistent with the present results.

## Experiment 2: The Effect of Mnemonic Digits Alone – No Impact on Temporal Processing

We demonstrated a perceptual/concurrent digit-induced NTA in Experiment 1. However, it is not yet clear whether a similar effect existed for mnemonic digits which disappeared before time encoding but were actively maintained in WM, i.e., a mnemonic digit-induced NTA. In order to investigate this point, we conducted Experiment 2 with a dual-task paradigm to explore whether the numerical magnitude kept in WM can modulate subsequent temporal processing. Participants were required to memorize a number and then reproduce the standard duration after perceiving it.

### Methods

#### Participants

A new sample of 21 participants (18 females and 3 males; mean age 20) were recruited and paid for Experiment 2.

#### Stimuli and Design

In Experiment 2, a dual-task paradigm was used, consisting of a number memorization task and a time reproduction task. Participants were first asked to memorize a small (1 or 2) or a large (8 or 9) number. Then, a time reproduction task was conducted. The reproduction task was similar to that in Experiment 1 except that the number at time perception stage was replaced by a black cross (1.5° × 1.5°) (see the second row of **Figure 1**). Participants were required to input the mnemonic digit by pressing an appropriate number key on the keyboard at the end of a trial. Each block of the experiment contained two repetitions of eight combinations of mnemonic digits (1, 2, 8, or 9) and standard durations (450 or 750 ms). The order of trials was randomized within a block. There were 10 blocks in this experiment, resulting in 160 trials in total. Participants took a rest after every two blocks.

#### Procedure

Trials once again began with a prompting sentence, followed by a 200 ms fixation cross and then an 800 ms blank. A mnemonic digit was then presented for 1000 ms. Participants were told to memorize this number. Five hundred milliseconds after the offset of the mnemonic digit, a black cross was presented for one of the two standard durations. A cue word “NOW” appeared on the screen and was replaced immediately by a green dot when participants pressed down the ‘0’ key to reproduce the standard duration. After completing the time reproduction task, participants input the mnemonic digit by pressing an appropriate number on the keyboard. The next trial began immediately after participants finished number input (see the second row of **Figure 1**).

### Results and Discussion

We analyzed all trials in which participants correctly recalled the mnemonic digit and produced reproductions that were longer than 1/3 of the standard duration and shorter than three times of the standard duration. With this trimming approach, 7.750% of the data (5.094% due to the distorted time reproduction and additional 2.656% due to incorrect memory performance) were removed as outliers. This trimming criterion for the dual-task paradigm was used throughout all the dual-task experiments of the present study. One participant’s data were removed due to a too low accuracy (25%) in the memory task.

#### Memory Accuracies

Memory accuracies were analyzed with a 2 (standard durations: short or long) × 2 (numerical magnitude of the mnemonic digit: small or large) repeated-measures ANOVA. Neither the two main effects nor the interaction was significant, *p*s > 0.10. This result suggested that none of the factors had an influence on the memory accuracy in this experiment.

#### Reproduced Durations

**FIGURE 3:**
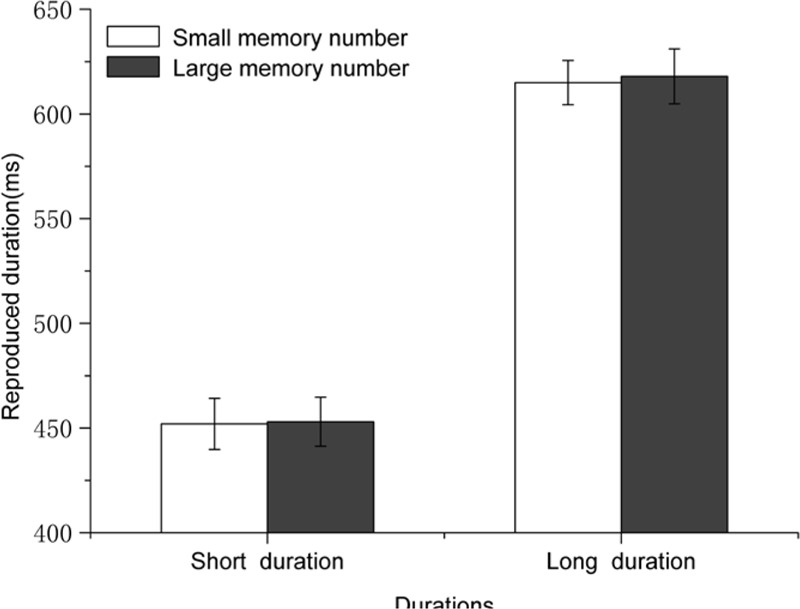
**Results for Experiment 2: the magnitude of the number stored in WM alone had no impact on time reproduction.** Error bars are within-subjects SEs (Cousineau, 2005).

Reproduced durations were also analyzed with a 2 (standard duration: 450 ms vs. 750 ms) × 2 (numerical magnitude of the mnemonic digit: small vs. large) repeated-measures ANOVA. The main effect of the standard duration was significant [*F*(1,19) = 53.52, *p* < 0.001, η^2^ = 0.738], indicating that participants reproduced a longer duration for the longer standard duration (617 ± 233 ms vs. 453 ± 164 ms). The main effect of the mnemonic digit was not significant [*F*(1,19) = 0.263, *p* = 0.614], suggesting that the reproduced durations were similar no matter whether the magnitude of the mnemonic digit was large or small (see **Figure 3**). Thus, magnitude of the mnemonic digit had no impact on temporal processing when the standard duration was presented with a black cross. The interaction between standard duration and magnitude of the mnemonic digit was not significant either [*F*(1,19) = 0.077, *p* = 0.784].

This result indicated that the magnitude of the number stored in WM could not affect temporal processing automatically and directly. In other words, we did not find a mnemonic digit-induced NTA in this experiment. It seemed that the magnitude information kept in WM could not access and modify the contents of the reference memory directly and influence subsequent temporal processing. A previous study (Sheth and Shimojo, 2003) demonstrated that the effects of postsensory mnemonic processing triggered by perceptually strong or weak targets were relatively weak, and equivalent only to the effects of perceptual processing triggered by perceptually weak, non-salient targets. According to this finding, it was likely that compared with processing triggered by the perceptual/concurrent digits in Experiment 1, processing triggered by the mnemonic digits in Experiment 2 of the present study was too weak to access the contents of the reference memory. If this were correct, a new paradigm with a sufficiently salient event, i.e., a task-irrelevant, perceptual/concurrent constant number (such as ‘5’), at the stage of time encoding might trigger strong processing to boost the accessibility of numerical magnitude stored in WM to the reference memory. This boosting effect might lead to a mnemonic digit-induced NTA. Meanwhile, since the perceptual/concurrent number ‘5’ in the new paradigm provided non-varying magnitude information, it would not produce a perceptual/concurrent digit-induced NTA as that in Experiment 1.

## Experiment 3: The Effect of Mnemonic Digits and Number ‘5’– An Impact Through Perceptual/Concurrent Digits

To verify the above hypothesis, we carried out Experiment 3 which was similar to Experiment 2 except that the black cross was replaced by a task-irrelevant perceptual/concurrent digit, i.e., a constant number ‘5.’ If our speculation above is correct, reproduced durations should be modulated by the magnitudes of the mnemonic digits.

### Methods

#### Participants

Another set of 26 participants (21 females and 5 males; mean age 19.15) were recruited and paid for Experiment 3.

#### Stimuli and Design

Stimuli and the design were the same as those in Experiment 2 except that the black cross in Experiment 2 was replaced by a constant number ‘5,’ which is has a magnitude in the middle of the mnemonic digit series (1, 2, 8, and 9), and provides only magnitude information at an average level (benchmark value) (see the third row of **Figure 1**).

#### Procedure

The procedure was identical to that in Experiment 2.

### Results and Discussion

The same trimming approach as that in Experiment 2 was used and 6.490% of the data (3.341% due to the distorted time reproduction and 3.149% due to incorrect memory performance) were removed as outliers in this experiment.

#### Memory Accuracies

A two-way repeated measure ANOVA was conducted with standard durations (short vs. long) and magnitudes of the mnemonic digits (small vs. large) as independent variables. Similar to Experiment 2, neither the main effect of the standard durations nor the main effect of the magnitude of the mnemonic digit was significant, *p*s > 0.10. The interaction between standard duration and magnitude of the mnemonic digit was not significant either, *p* > 0.10. Memory accuracy was thus not influenced by any factor in this experiment.

#### Reproduced Durations

**FIGURE 4:**
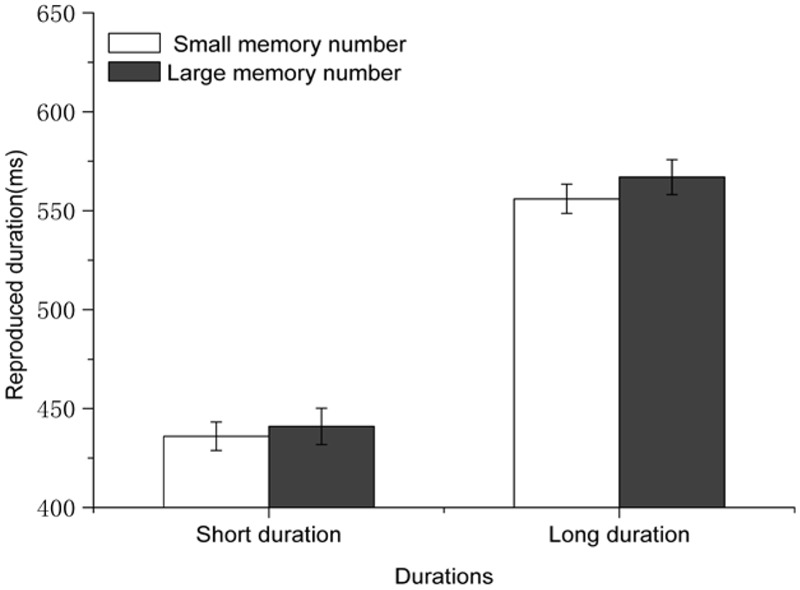
**Results for Experiment 3: Magnitude of the number stored in WM affected time reproductions when the standard duration was represented by a constant number 5 (benchmark value).** Error bars are within-subjects SEs (Cousineau, 2005).

A 2 (Magnitude of the mnemonic digit: small vs. large) × 2 (Duration: short vs. long) repeated measures ANOVA was performed on the reproduced durations across all participants. The interaction between standard duration and magnitude of the mnemonic digit was not significant [*F*(1,25) = 0.720, *p* = 0.404, η^2^ = 0.028]. The results revealed a significant main effect of standard durations [*F*(1,25) = 66.404, *p* < 0.001, η^2^ = 0.726], suggesting that long standard durations were reproduced longer than short durations (561 ± 153 ms vs. 438 ± 139 ms). Most importantly, the main effect of magnitude of the mnemonic digit was significant [*F*(1,25) = 5.230, *p* = 0.031, η^2^ = 0.173], indicating that in the condition in which the temporal target contained a constant number, participants reproduced longer durations when they kept a large number in memory than when they kept a small number in memory (504 ± 140 ms vs. 495 ± 145 ms), i.e., a mnemonic digit-induced NTA (see **Figure 4**).

Thus, the magnitude of the mnemonic digit had an impact on subsequent time reproduction when the target of the time reproduction contained a constant digit. In other words, the magnitude representations of the mnemonic digits actively kept in WM can modulate subsequent time processing, i.e., the mnemonic digit-induced NTA. Here, we have demonstrated a second type of NTA (different from the perceptual/concurrent digit-induced NTA) in a time reproduction task which is a direct assessment of perceived duration and is free of potential decision-making bias (Yates et al., 2012; Rammsayer and Verner, 2014). This finding provides supporting evidence for the proposal of an earlier study (Vicario, 2011) that participants were performing implicit comparisons of size differences across the displayed numbers when those numbers were all randomly arranged in the same block (intermingled design). It is noteworthy that the magnitude of the mnemonic digit-induced NTA found in the present study (Mean difference of time reproductions between large and small digits = 9 ms) was in the same range as the magnitude of the perceptual/concurrent digit-induced NTA found in Experiment 1 of the present study (Mean difference of time reproductions between large and small digits = 12 ms) and in a previous study (Cai and Wang, 2014, Experiment 1, Mean difference of time reproductions between large and small digits = 13 ms).

Taking the results of Experiments 2 and 3 together, it is clear that perceptual/concurrent digits are necessary for the modulating effect of mnemonic digits on temporal processing. It appears that the NTA actually relied on a perceptual number-time co-occurrence at the stage of time encoding. Without it, numerical magnitudes of either the perceptual/concurrent digits or the mnemonic digits could not influence participants’ performance in the temporal reproduction task. Thus, the mnemonic digit-induced NTA was likely due to an indirect modulation of mnemonic digits on time perception via the perceptual/concurrent digits.

## Experiment 4: A Congruency Effect Between Perceptual/Concurrent and Mnemonic Digits

The above three experiments demonstrated the existence of two types of NTAs, one induced by perceptual/concurrent digits and the other by mnemonic digits. These results implied that a perceptual number-time co-occurrence at the target encoding, i.e., an association between the numerical representations of perceptual/concurrent digits and the reference memory, built up during processing of the standard duration. Through the number-time co-occurrence, the contents of the reference memory could be accessed and modified by both sensory and postsensory numerical magnitudes. If both the perceptual and mnemonic digit-induced NTAs relied on this perceptual number-time co-occurrence, we would expect that the modulation effects of the perceptual and mnemonic digits on temporal reproduction can cancel each other out in certain situations. We tested this hypothesis with a new paradigm combining components used to explore the perceptual/concurrent digit-induced NTA in Experiment 1 and the mnemonic digit-induced NTA in Experiment 3. In this dual-task paradigm, a mnemonic digit was presented as a WM task, as in Experiment 3, and a task-irrelevant perceptual/concurrent digit was presented during the stage of time perception, as in Experiment 1. Participants were required to memorize the mnemonic digit and then reproduce the standard duration consisting of the perceptual/concurrent digit. We tried to explore whether WM contents could modulate the NTA when the magnitude of the mnemonic digit and that of the perceptual/concurrent digit were congruent (both were large or small numbers) or incongruent (one small and one large number). This experiment aimed to deepen our understanding of how the two types of NTAs are related.

### Methods

#### Participants

A new set of 19 participants (18 females and 1 male; mean age 20.21) were recruited and paid for this experiment.

#### Stimuli and Design

In this experiment, a dual-task paradigm, consisting of a number memorization task and a time reproduction task, was used. Participants were asked to memorize a small (1, 2) or a large (8, 9) number first (as in Experiment 3). Then, a time reproduction task (identical to that in Experiment 1) was conducted. Participants were required to input the mnemonic digit by pressing an appropriate number key on the keyboard at the end of each trial. The small/large magnitude of the mnemonic digits in the WM task and the small/large magnitude of the perceptual/concurrent digits in the time reproduction task generated two critical experimental conditions: congruent and incongruent conditions. The congruent condition referred to a trial in which both magnitudes of the mnemonic digit and the perceptual/concurrent digit were small or large. The incongruent condition referred to a trial when one of the memorized or the perceptual/concurrent digits was a small number (1 or 2), whereas the other was a large number (8 or 9). It should be noted that the mnemonic and the perceptual/concurrent digits were never identical in the congruent condition of the present study. Instead, they were related at an abstract level, i.e., belonging to the same magnitude category. For example, if both digits were large numbers, then one digit would be 9 and the other would be 8. Similarly, if both perceptual/concurrent and mnemonic digits were small numbers, then one digit would be 1 and the other would be 2. This stimulus arrangement was an important departure from a previous study (Bi et al., 2014) and ensured that any effect found in the present study could not be attributed to a mechanism based on ‘complete match’ between the mnemonic and the perceptual/concurrent digits (Bi et al., 2014).

Each block of the experiment contained two repetitions of 16 combinations of the perceptual/concurrent digits (1, 2, 8, and 9), congruency (congruent or incongruent) and standard durations (450 or 750 ms). The order of trials was randomized within a block. There were 10 blocks in this experiment, resulting in 320 trials in total. Participants took a rest after every two blocks.

#### Procedure

Trials began with a prompting sentence, followed by a 200 ms fixation cross and then an 800 ms blank. A mnemonic digit was then presented for 1000 ms. Participants were told to memorize this number. Five hundred milliseconds after the offset of the mnemonic digit, a digit was presented for one of the two standard durations. A cue word “NOW” appeared on the screen and was replaced immediately by a green dot when participants pressed down the ‘0’ key to reproduce the standard duration. After completing the time reproduction task, participants input the mnemonic digit by pressing an appropriate number on the keyboard. The next trial began immediately after participants finished number input (see the fourth row of **Figure 1**).

### Results and Discussion

The same trimming approach as that used in Experiment 2 was used and 5.378% of the data (1.776% due to the distorted time reproductions and 3.602% due to incorrect memory performance) were removed as outliers. Memory accuracies and reproduced durations of the standard durations were analyzed separately.

#### Memory Accuracies

Memory accuracies were analyzed with a 2 (standard duration: 450 ms vs. 750 ms) × 2 (congruency: congruent vs. incongruent) × 2 (magnitude of the perceptual/concurrent digit: small vs. large) repeated-measures ANOVA. Results showed that there were no significant main effects or interactions between these factors, *p*s > 0.10.

#### Reproduced Durations

**FIGURE 5:**
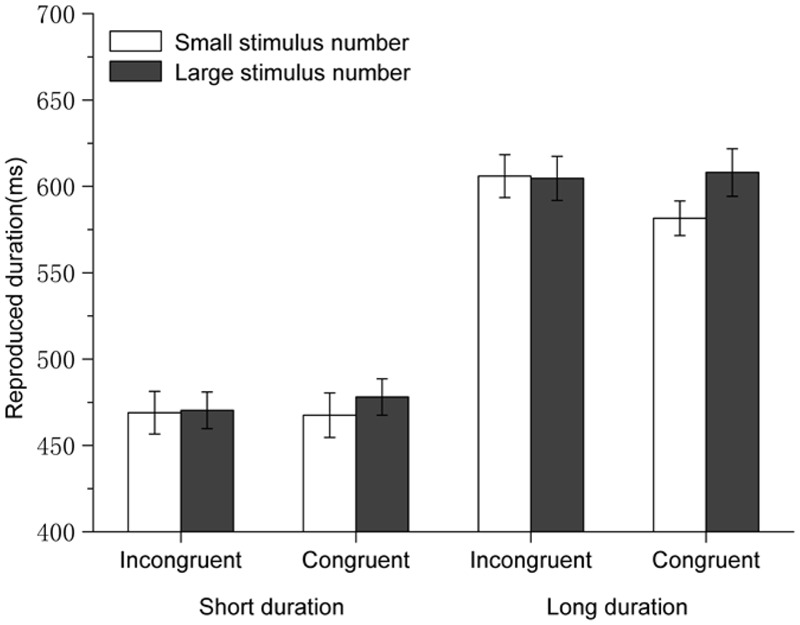
**Results for Experiment 4: participants tended to reproduce standard durations with large perceptual/concurrent digits longer than those with small perceptual/concurrent digits in the congruent condition but not in the incongruent condition.** Error bars are within-subjects SEs (Cousineau, 2005).

Reproduced durations were subjected to a 2 (standard duration: 450 or 750 ms) × 2 (congruency: congruent or incongruent) × 2 (numerical magnitude of the perceptual/concurrent digit: small or large) repeated-measures ANOVA. Results showed that the main effect of standard duration was significant, [*F*(1,18) = 36.129, *p* < 0.001, η^2^ = 0.667] in that the longer durations were reproduced longer than the short durations (600 ± 207ms vs. 471 ± 173 ms). The main effect of the numerical magnitude of the perceptual/concurrent digit, the two-way interaction between the standard duration and congruency, and the two-way interaction between standard duration and numerical magnitude of the perceptual/concurrent digit were not significant (*p*s > 0.05). Critically, the interaction between numerical magnitude of the perceptual/concurrent digit and congruency was significant [*F*(1,18) = 4.618, *p* = 0.045, η^2^ = 0.204]. When both of the mnemonic digit and perceptual/concurrent digit were small numbers or large numbers, reproductions of standard durations were longer when the perceptual/concurrent digit was a large number than when it was a small number (543 ± 194 ms vs. 524 ± 173 ms, difference = 19 ms, *p* = 0.02) (see **Figure 5**). However, when one of the two digits was a small number and the other one was a large number, reproduced durations were similar for large perceptual/concurrent digits and small perceptual/concurrent digits (*p* > 0.5). The three-way interaction between these three factors was not significant, *p* > 0.10.

The results showed that WM processing did modulate the NTA. Critically, this modulation depended on the congruency between the magnitude of the mnemonic digit and the magnitude of the perceptual/concurrent digit. The NTA was observed when the magnitude of the mnemonic digit was congruent with the magnitude of the perceptual/concurrent digit (both were large or small numbers), but not when the magnitude of the mnemonic digit was incongruent with the magnitude of the perceptual/concurrent digit (one small and one large number). These results provide evidence for the view that the two types of the NTAs are closely related with each other. Specifically, the effects of the perceptual/concurrent and the mnemonic digits on temporal reproduction canceled each other out.

## Experiment 5: The Congruency Effect – Necessity of Working Memory

There could be another explanation for the modulation on the NTA in Experiment 4. Previous studies demonstrated that numbers, especially Arabic numerals, can be automatically converted into quantities (Dehaene and Akhavein, 1995; Koechlin et al., 1999; Ito and Hatta, 2003). The interference effect in Experiment 4 might be due to an automatic activation (via priming a digit with mere exposure but no memory requirement) rather than the memorization of the mnemonic digit. In order to investigate whether the modulation effect on the NTA was induced by the automatic activation/priming or by a memory-based effect, we carried out Experiment 5. The same paradigm and procedure as those used in Experiment 4 were used in Experiment 5 except that participants were not required to memorize and input the first number (the mnemonic digit in Experiment 4) in each trial.

### Methods

#### Participants

A new sample of 21 participants (18 females and 3 males; mean age: 20.86) were recruited and paid for this experiment.

#### Stimuli and Design

Stimuli and the design were identical to those in Experiment 4.

#### Procedure

The procedure was similar to that in Experiment 4 with an exception: participants were asked to attend to the first number (the mnemonic digit in Experiment 4), but they were not required to memorize it, and there was no recall of this number at the end of each trial (see the fifth row of **Figure 1**).

### Results and Discussion

**FIGURE 6:**
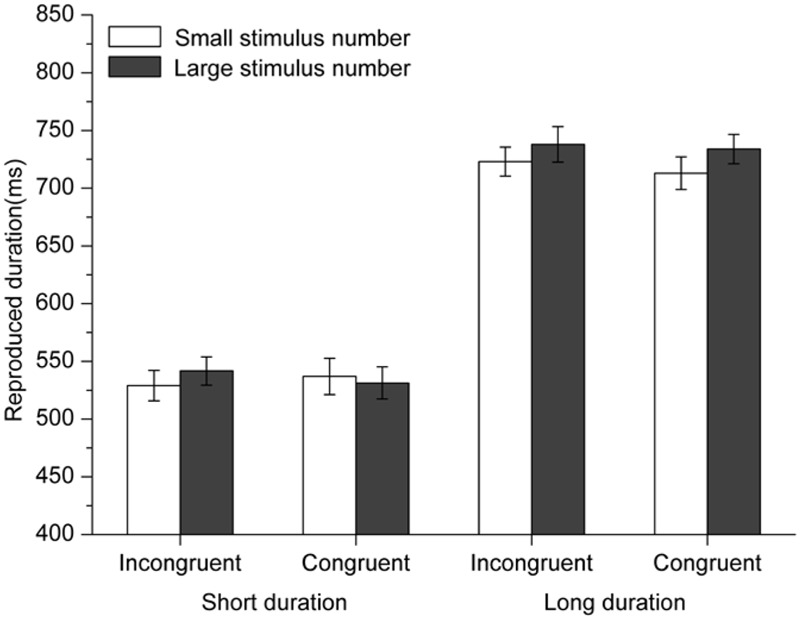
**Results for Experiment 5: priming a digit with mere exposure but no memory requirement could not distort the NTA, no matter whether its magnitude was congruent or incongruent with the magnitude of the perceptual/concurrent digit.** Error bars are within-subjects SEs (Cousineau, 2005).

The same trimming approach as that in Experiment 1 was used and 2.140% of the data were excluded as outliers. A three-way repeated-measures ANOVA was performed with standard duration (450 vs. 750 ms), congruency (congruent vs. incongruent), and numerical magnitude of the perceptual/concurrent digit (small vs. large) as independent variables. The main effect of the numerical magnitude of the perceptual/concurrent digit [*F*(1,20) = 12.3, *p* = 0.002, η^2^ = 0.381] was significant, suggesting that those standard durations with large numbers were reproduced longer (626 ± 187 ms vs. 637 ± 186 ms for the two number magnitudes). The interaction between standard duration and numerical magnitude of the perceptual/concurrent digit also reached significance [*F*(1,20) = 5.623, *p* = 0.028, η^2^ = 0.219]. Simple main effects analysis showed that numerical magnitude of the perceptual/concurrent digit affected temporal processing for long durations (736 ± 216 vs. 718 ± 210, difference = 18 ms, *p* < 0.001), but not for short durations (*p* = 0.462). Similar to the results in Experiment 4, the ANOVA revealed a significant main effect of standard duration [*F*(1,20) = 56.652, *p* < 0.001, η^2^ = 0.739], indicating that longer standard durations were reproduced longer (535 ± 213 ms vs. 727 ± 173 ms for the two standard durations). However, unlike the results in Experiment 4, the interaction between numerical magnitude of the perceptual/concurrent digit and congruency was not significant [*F*(1,20) = 0.604, *p* = 0.446]. This suggests that the presentation of the first number (the mnemonic digit in Experiment 4) without memory requirement had no impact on the NTA, no matter whether its magnitude was congruent with the magnitude of the perceptual/concurrent digit or not (see **Figure 6**). The three-way interaction between these factors did not reach significance either, *p* > 0.10.

Taking Experiments 4 and 5 together, WM contents in the form of the magnitude of the mnemonic digit, as well as sensory contents in the form of the magnitude of the perceptual/concurrent digit, appear to be able to modulate time processing simultaneously. The effect of numerical magnitude on temporal processing was abolished when the magnitude of the perceptual/concurrent digit was incongruent with the magnitude of the number that stored in WM (in Experiment 4). Memory processing was a necessary component for the modulation of WM on perceptual/concurrent digit-induced NTA (in Experiment 5).

## General Discussion

Our study contributed to the idea that temporal processing is profoundly influenced by both sensory and postsensory task-irrelevant numerical magnitudes. In contrast to digits of small magnitude, such as 1 and 2, digits of large magnitude, such as 8 and 9, expanded the perceived duration of a target interval and led to an increased temporal reproduction, either when they were presented concurrently with the target interval or when they were presented well before the target interval (where a constant number ‘5’ was shown serving as a benchmark value) and actively maintained in WM. Our study demonstrates the existence of a mnemonic digit-induced NTA. Most importantly, we showed how the new type of NTA was closely linked with the traditional perceptual/concurrent digit-induced NTA. The close relationship between the two types of NTAs was revealed in two contexts. First, although we demonstrated that a digit which was out of perception and actively kept in WM distorted a subsequent temporal reproduction, this particular modulation effect was contingent on a perceptual number-time co-occurrence at the stage of time encoding. Second, the impact of numerical magnitudes, in the forms of perceptual/concurrent digits and mnemonic digits, on temporal processing canceled each other out, leading to a congruency effect of numerical magnitudes. Below, we attempt to extend and discuss the implications of our findings in the context of the current literature.

The present study demonstrated that temporal representations stored in the reference memory were accessed and modified by task-irrelevant sensory or postsensory numerical magnitudes. These findings are consistent with previous studies reporting many interference effects of various non-temporal magnitude dimensions on concurrent temporal processing. For example, a duration was judged longer subjectively if it was larger in size (Xuan et al., 2007; Alards-Tomalin et al., 2014; Rammsayer and Verner, 2014), consisted of more items (Mo and Michalski, 1972; Long and Beaton, 1981; Dormal et al., 2006; Xuan et al., 2007; Hayashi et al., 2013), had a higher intensity (Berglund et al., 1969; Ekman et al., 1969; Goldstone et al., 1978; Matthews et al., 2011), had a higher speed (Kanai et al., 2006), contained stimuli with a longer line (Casasanto and Boroditsky, 2008), presented in the right hemispace (Vicario et al., 2008), or occupied a body gesture covering longer spatial distance (Cai et al., 2013). Traditionally, those effects were explained in a theoretic framework of commonly shared representational system among various magnitude dimensions, including time, space and quantity. Specifically, Walsh (2003) proposed an ATOM (A Theory of Magnitude) theory to explain the interactions among time, space and quantity. According to the ATOM theory, time, space and quantity share a common origin of a generalized magnitude system. Thus, these different dimensions of magnitude, sharing common cognitive and neural mechanism, can interfere with each other during concurrent processing (Walsh, 2003). Our finding of a mnemonic-digit induced NTA add new evidence to this theoretic framework by suggesting that this generalized magnitude system may receive inputs from different stages/aspects of cognitive processing, including perception and WM, simultaneously. More importantly, the contributions of perceptual and mnemonic processing to this generalized magnitude system may be interchangeable at a certain level- leading to a cancelation effect of incongruent perceptual and mnemonic magnitudes.

The results of Experiments 1–3 revealed that the perceptual/concurrent and mnemonic digits modulated temporal processing in different ways. The necessity of the perceptual number-time co-occurrence for the mnemonic type NTA is consistent with previous research (Sheth and Shimojo, 2003, p. 181) suggesting “that memory is at best, only a faint echo of the actual percept and never the equivalent of a vivid, strong percept.” According to this idea, it is very likely that the mnemonic representations induced by mnemonic digits were not strong enough to exert a direct influence on temporal magnitude in the reference memory. However, during the presentation of the task-irrelevant number ‘5’ which was perceptually salient and strong, the accessibility of the numerical information of the mnemonic digits to the reference memory was boosted at a certain level. This boosting effect on the mnemonic digits, via the perceptual number-time co-occurrence, led to modifications of the temporal representations in the reference memory, i.e., a mnemonic digit-induced NTA.

The exact mechanism of the boosting effect is not yet clear. Here, we tentatively propose that one potential candidate might be related to cognitive control, which has been defined as “the ability to regulate, coordinate, and sequence thoughts and actions in accordance with internally maintained behavioral goals” (Braver, 2012, p. 106). According to Braver et al. (2007), cognitive control involves two operating modes or strategies, i.e., ‘proactive control’ and ‘reactive control.’ Proactive control reflects the sustained and anticipatory maintenance of goal-relevant information to enable optimal cognitive performance. On the other hand, reactive control reflects transient stimulus-driven goal reactivation based on interference demands or episodic association. In other words, proactive control invokes the anticipation and prevention of interference before it occurs, whereas reactive control depends on the detection and resolution of interference after its onset (Braver et al., 2007; Braver, 2012).

Here, we tentatively suggest that it was very likely that different task requirements across Experiments 1–3 of the present study led to varying balances of the two control strategies. For example, the perceptual/concurrent digits along with the standard duration in Experiment 1 were sufficiently salient events (relative to the mnemonic numerical representations in Experiment 2) which could trigger some bias favoring the reactive control strategy over the proactive control strategy. The reactive control strategy may be the default control mode for this paradigm since there was no advance contextual cues before the onset of the standard duration to elicit strong anticipation and prevention of upcoming interference. Under the reactive control strategy, task-irrelevant information, i.e., the numerical representations, was not actively inhibited (relative to that under the proactive control strategy) and was still active during the processing of the standard duration, leading to a typical NTA effect in Experiment 1. In contrast, there were two task-defined goals in Experiment 2, i.e., the memorization of the mnemonic digit (Task 1) and the reproduction of the standard duration (Task 2). The processing of the mnemonic digits occurred well before the processing of the time reproduction, rendering the information of the numerical representations (produced by the Task 1) irrelevant to the Task 2, i.e., the time reproduction. Thus, a proactive control was more likely to be applied before the onset of the standard duration in order to optimize preparation to the Task 2 while to minimize interference from internal or external sources of distraction, such as the Task 1-relevant (but Task 2-irrelevant) numerical information. This may partially explain why the mnemonic numerical representations were inhibited more successfully during the processing of the time reproduction and there was no significant NTA effect in Experiment 2. Furthermore, Experiment 3 introduced a task-irrelevant digit ‘5’ along with the standard duration. The digit ‘5’ in Experiment 3 triggered a shift in the relative utilization from reactive to proactive control strategy and showed a similar effect as the perceptual digits in Experiment 1. Thus, the numerical information induced by the mnemonic digits in Experiment 3 was not actively inhibited (relative to that in Experiment 2), leading to a mnemonic digit-induced NTA in Experiment 3.

Moreover, our Experiments 4 and 5 revealed a congruency effect of numerical magnitudes (in the forms of perceptual and mnemonic representations) on temporal processing. An interesting issue regarding the characteristic of this congruency effect concerns the level, perceptual or mnemonic, at which it originates. Previous studies have demonstrated that regardless of whether participants had to explicitly remember or simply attend to non-temporal magnitudes, such as digits (Cai and Wang, 2014) or spatial distances (Casasanto and Boroditsky, 2008), they exhibit a typical perceptual type of the NTA to equivalent extents. Thus, the perceptual type of NTA does not rely on memorization processing and the locus of the congruency effect is not clear. An alternative explanation to the congruency effect is that the congruency effect is due only to perceptual processing of the two digits, i.e., without a memorization component involved. However, we did not observe a congruency effect in Experiment 5, suggesting that the congruency effect operated at a mnemonic level, possibly in the reference memory or in WM. In this sense, our results also implied a close relationship between WM and the reference memory (Zakay and Block, 1997; Jones and Wearden, 2004).

Other perceptual/concurrent stimulus-induced associations between quantity and time have also been observed in various non-symbolic dimensions, such as non-symbolic numerosity (e.g., quantity of dots in the stimulus), volume of space (Delong, 1981), distance covered by gesture (Cai et al., 2013), number of items (Hayashi et al., 2013), length of stimulus (Casasanto and Boroditsky, 2008), intensity (Goldstone et al., 1978; Matthews et al., 2011), and weight (Lu et al., 2009). In order to extend our finding about the mnemonic stimulus-induced NTA into a wider scope, it will be necessary to explore in new studies whether mnemonic magnitudes of the non-symbolic numerosity can modulate subsequent time processing in a way similar to mnemonic magnitudes of the symbolic numerosity in the present study.

In Experiments 1 and 5, we found significant interactions between numerical magnitude and standard duration, i.e., the perceptual/concurrent digit-induced NTA effect was modulated by the length of the standard duration. It could be that the perceptual/concurrent digit-induced NTA in Experiments 1 and 5 needed some time (750 ms but not 450 ms) to be fully developed during the encoding of a standard target interval. Similar interactions were also observed in previous studies with the same task (time reproduction) but a different stimulus dimension (sizes of stimuli) (Long and Beaton, 1980; Rammsayer and Verner, 2014, 2015). For example, Rammsayer and Verner (2015) demonstrated an interaction between reproduced duration and stimulus size in their study. They found that durations of larger stimuli were reproduced longer when the standard durations were 1200 ms but not when they were 800 ms or 1000 ms (Rammsayer and Verner, 2015). However, other studies showed that the interference effect of a non-temporal stimulus size on temporal processing was manifested when a target duration was as short as 70 ms or 50 ms (Cantor and Thomas, 1976; Thomas and Cantor, 1976). Furthermore, some other studies (Chang et al., 2011; Cai and Wang, 2014) with the same temporal task (time reproduction) and stimuli (numbers of different magnitudes) did not find such an interaction. Thus, the current literature provides mixed results upon this point. Those interactions induced by perceptual/concurrent stimuli seemed to be volatile and require further investigation. Another interesting observation in the present study is that the interaction between numerical magnitude and standard duration did not reach significance in Experiment 3 when a mnemonic digit-induced NTA was observed, i.e., the mnemonic digit-induced NTA was not modulated by the standard durations (the durations of the constant number ‘5’). This is not surprising since a critical departure of Experiment 3 from Experiment 1 is that the constantly changing magnitudes (mnemonic digits) and the target durations were presented asynchronously. Thus, the representations of the constantly changing (mnemonic) digits in Experiment 3 were well processed and built up before the start of the encoding of the standard durations, leaving no or limited space for the modulation of the standard durations on the NTA. Further studies should be conducted to explore this topic and investigate the potential boundary conditions and the underlying mechanisms of such an interaction for the perceptual/concurrent digit-induced NTA as well as for the mnemonic digit-induced NTA.

A number of studies have been conducted to investigate the neurophysiological mechanism of the NTAs. An event-related potential (ERP) study (Xuan et al., 2009) showed that in a time comparison task, P1 and the contingent negative variations (CNVs) corresponding to the first duration and the second duration were all enhanced when perceptual/concurrent digits were small, suggesting that the NTA occurred at a relatively early stage of perceptual level. Hayashi et al. (2013) explored the interaction between numerosity and time with functional magnetic resonance imaging (fMRI) and transcranial magnetic stimulation (TMS). They found that numerosity interacted with temporal processing at two stages in different brain regions. At a perceptual level, the interaction occurred in the parietal region and at a categorical decision level, the interaction occurred in the prefrontal cortex. These studies shed light on the precise mechanism of NTA through approaches with superior temporal resolution or spatial resolution.

Taken together, the experimental results of our study shed new light on the mechanism of the NTA. We have demonstrated that typical associations between number and time (NTA) include at least two types, one induced by perceptual/concurrent digits and the other induced by mnemonic digits. Our study also revealed that the sensory and postsensory contributions to the NTA were related and could operate simultaneously. Further research is needed to underpin the underlying neural mechanism of how sensory and postsensory processing contribute to the NTAs from different perspectives with multiple neuroscience approaches such as ERP and fMRI.

## Ethics Statement

All procedures performed in studies involving human participants were in accordance with the ethical standards of the institutional and/or national research committee, the American Psychological Association (APA) standards and with the 1964 Helsinki declaration and its later amendments or comparable ethical standards.

## Author Contributions

All of the authors contributed to the design of the study and preparation of the manuscript. GJ acquired the data and analyzed it with ZF.

## Conflict of Interest Statement

The authors declare that the research was conducted in the absence of any commercial or financial relationships that could be construed as a potential conflict of interest.

## Footnotes

1 It is worth to note that the present study emphasized on the temporal processing at a time scale of sub-second level since a previous study (Lewis and Miall, 2003) had revealed that the mechanisms of visual estimations between sub-second and supra-second levels are different and the sub-second temporal reproduction may involve more automatic processing.

2 *Post hoc* analyses in each ANOVA of each experiment were conducted using the Bonferroni correction for multiple comparisons.
